# Mitochondrial F-ATP Synthase and Its Transition into an Energy-Dissipating Molecular Machine

**DOI:** 10.1155/2019/8743257

**Published:** 2019-04-15

**Authors:** Giovanna Lippe, Gabriele Coluccino, Marco Zancani, Walter Baratta, Paola Crusiz

**Affiliations:** Department of Agricultural, Food, Environmental and Animal Sciences, University of Udine, Italy

## Abstract

The mitochondrial F-ATP synthase is the principal energy-conserving nanomotor of cells that harnesses the proton motive force generated by the respiratory chain to make ATP from ADP and phosphate in a process known as oxidative phosphorylation. In the energy-converting membranes, F-ATP synthase is a multisubunit complex organized into a membrane-extrinsic F_1_ sector and a membrane-intrinsic F_O_ domain, linked by central and peripheral stalks. Due to its essential role in the cellular metabolism, malfunction of F-ATP synthase has been associated with a variety of pathological conditions, and the enzyme is now considered as a promising drug target for multiple disease conditions and for the regulation of energy metabolism. We discuss structural and functional features of mitochondrial F-ATP synthase as well as several conditions that partially or fully inhibit the coupling between the F_1_ catalytic activities and the F_O_ proton translocation, thus decreasing the cellular metabolic efficiency and transforming the enzyme into an energy-dissipating structure through molecular mechanisms that still remain to be defined.

## 1. Introduction

Mitochondria are highly dynamic enclosed organelles harbouring an outer membrane (OMM) and an inner membrane (IMM) with a small intermembrane space separating them. The surface of the IMM is significantly bigger than that of the OMM due to the presence of numerous invaginations called cristae that extend more or less deeply into the protein-dense central matrix [1]. In differentiated aerobic cells, mitochondria are crucial for ATP production from nutrient oxidation; for ROS (reactive oxygen species) production, which contributes to mitochondrial damage in several pathologies and to redox signalling from the organelle to the rest of the cell [2, 3]; for intracellular calcium signalling; and for execution of cell death among other functions [4]. This functional versatility is matched by their great variability in number and structure depending on the tissue and the developmental stage. Mitochondria interact with the cytoskeleton, which modulates their subcellular localization and motility, and with the endoplasmic reticulum for calcium homeostasis. ATP is produced from ADP and phosphate (Pi) by the F-type ATP synthase complex (or complex V) in a process known as oxidative phosphorylation, which takes place in the IMM. The four complexes of the respiratory chain carry out a series of redox reactions, resulting in oxygen reduction to water, which are able to sustain the proton-pumping activity of complexes I, III, and IV. These latter generate an electrochemical gradient across the IMM known as proton motive force, which is absolutely necessary for F-ATP synthase to produce ATP [5]. From the intermembrane space, however, protons may leak back to the mitochondrial matrix independent of ATP synthesis, decreasing the metabolic efficiency and giving rise to mitochondrial uncoupling. In the last decade, there has been a growing interest in characterizing the endogenous dissipating pathways, as well as in the chemical agents able to induce a mild mitochondrial uncoupling, which may provide a powerful therapeutic treatment for widespread diseases such as obesity and diabetes [6, 7]. This review is especially dedicated to F-ATP synthase and its transition into an energy-dissipating enzyme through molecular mechanisms that still remain to be defined.

## 2. F-Type ATP Synthase as a Molecular Motor

The complex structure and the unique functional mechanism of F-ATP synthase are now known thanks to more than 50 years of studies by several researchers, including the three scientists that were awarded the Nobel Prize: Sir Peter Mitchell, who demonstrated that F-ATP synthase relies on the electrochemical gradient to perform catalysis [5]; Sir John E. Walker, who solved the mammalian F-ATP synthase structure [8]; and Paul Boyer, who clarified the mechanism of rotational catalysis [9]. Nevertheless, some aspects of the coupling mechanism between proton translocation and catalysis remain to be fully understood [10].

In all energy-converting membranes, F-ATP synthase consists of a roughly globular, water-soluble F_1_ head and a membrane-embedded F_O_ subcomplex comprising the *a* subunit and a ring of multiple *c* subunits. These moieties are connected by two stalks: the lateral or peripheral stalk, which is structurally part of the F_O_ moiety, and the central stalk, which is associated to the F_1_ sector [10]. All types of F-ATP synthases function as nanometer-scale rotary machines consisting of two motors linked by a rotor, which comprises the *c*-ring and the central stalk. One motor located in the F_O_ sector generates movement of the rotor at a rate of ~100 revolutions/s by consuming the proton motive force; the other, located in the F_1_ moiety, uses energy transmitted by the rotor to synthesize ATP. The synthetic motor can work in reverse, driving the rotor backward with energy from ATP, releasing ADP and phosphate, and generating a membrane potential [1]. The strict correlation between proton flux and rotor rotation has been confirmed by measurement of the H^+^ : ATP ratio in a bacterial F-ATP synthase, where a “perfect chemomechanical coupling” between proton translocation, rotary motion, and ATP synthesis/hydrolysis has been reported, excluding “slip” of the rotor, i.e., rotation of the *c*-ring without carrying a proton [11]. This coupling is instead disrupted by the antibiotic oligomycin, which binds the *c*-ring, preventing it to rotate in either directions [12]. Our understanding of how the enzyme works has greatly come from single-molecule studies, which allowed direct visualization by fluorescence microscopy of rotating bacterial F-ATP synthases driven by ATP hydrolysis [13] and, more recently, from electron cryomicroscopy analysis of mitochondrial and bacterial ATP synthases, revealing the architecture of the F_O_ sector and thus the mechanisms of proton translocation [14].

In its simplest bacterial form, the F_O_ sector consists of 9-15 copies of the subunit *c*, two copies of the subunit *b*, and a copy of the subunit *a* [14]. While the *b* subunits constitute part of the peripheral stalk [15], the subunit *a* is embedded in the membrane, where it is organized into a four-helix horizontal bundle that wraps around the *c*-ring, forming two semichannels through which the H^+^ flow by protonation/deprotonation of conserved carboxylic residues present in each *c* subunit [16–18]. The eukaryotic F_O_ sector is made up of subunits A6L, *e*, *f*, and *g* and 2 or 3 other additional subunits (DAPIT and 6.8PL in vertebrates; *i*/*j*, *k*, and *l* in yeast), besides subunits *a*, *b*, and *c* [14]. The smaller *c*-ring, which comprises 8 or 10 *c* subunits in metazoans and yeast, lowers the bioenergetic cost of ATP synthesis, i.e., the H^+^/ATP stoichiometry from 3.3 to 2.7, respectively [19].

The peripheral stalk tends to diverge throughout evolution, even though the overall structure seems to be constant [15]. The bacterial peripheral stalk consists of the *b*_2_ dimer, which spans the whole enzyme, and a single copy of the subunit *δ*. Like the F_O_ membrane-embedded part, the eukaryotic peripheral stalk is more complex. Its membrane distal part is constituted by one copy of the subunits *b*, F6, *d*, and OSCP (oligomycin-sensitivity conferral protein, homologous to the bacterial *δ* subunit) [20], while its base comprises the C-terminal region of subunit A6L [18, 21], the N-terminal domain of the subunit *f* [18], and the subunit *i*/*j* in yeast [22].

**Figure 1:**
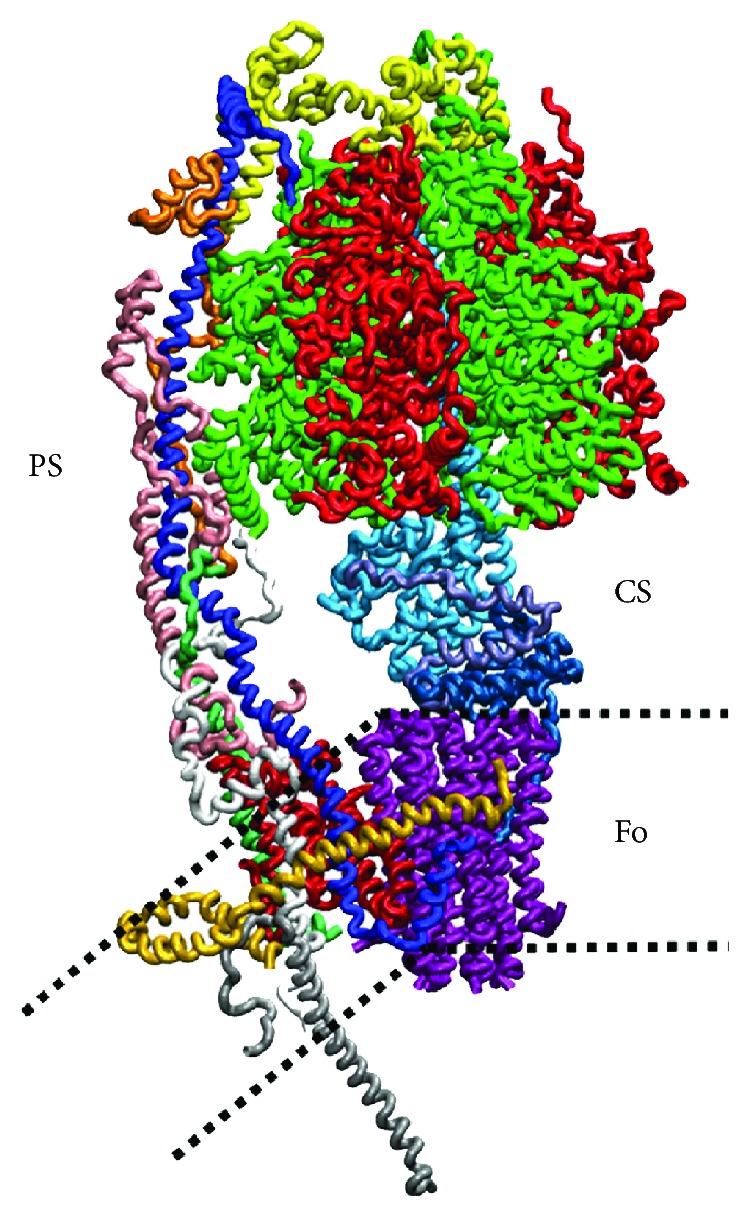
Structure of the mitochondrial F-ATP synthase. Subunits are shown in colors as follows. F_1_ is shown with the alternating *α* (green) and *β* subunits (red). On the left, the peripheral stalk (PS) includes the OSCP (yellow), *b* (dark blue), F6 (orange), and *d* (pink) subunits. The central stalk (CS) connecting the *α*_3_*β*_3_ subcomplex to the *c*-ring composed of 8 identical subunit *c* (purple) includes the *γ* (cyan), *δ* (blue), and *ε* (ice blue) subunits. The F_O_ membrane sector includes the subunits *a* (dark red, mostly covered in the picture by other subunits), *f* (white), A6L (emerald, mostly covered by other subunits), *g* (light orange), and *e* (silver). In the membrane region, which is delineated by dotted lines, the subunits *e* and *g*, with the N-terminal part of subunit *b*, create a subdomain that bends the inner mitochondrial membrane.

F_1_ is the catalytic sector, which is always composed of three *αβ* dimers that alternate in surrounding the central stalk. The latter comprises the *γ* subunit, which is associated with the subunit *ε* in bacteria and with the subunits *δ* and *ε* in eukaryotes [10] (Figure 1). Indeed, the bacterial *ε* subunit is homologous to the mitochondrial *δ* subunit, whereas the mitochondrial *ε* subunit has no equivalent in the bacterial enzyme [10]. The catalytic nucleotide binding sites are located in the three *β* subunits at the interfaces with the respective three *α* subunits. According to Boyer's catalysis model, subunit *γ* rotation within *α*_3_*β*_3_ takes each of the three *β* subunits through three major functional conformations, denoted *β*_E_ (empty), *β*_DP_ (bound to ADP), and *β*_TP_ (bound to ATP), thereby synthesizing three Mg^2+^-ATP molecules during each 360° rotation. During ATP hydrolysis, the transition between *β*_E_, *β*_TP_, and *β*_DP_ states drives the opposite rotation of the *γ* subunit and the *c*-ring, thereby causing the formation of a proton gradient. In both ATP synthetic and hydrolytic directions, Mg^2+^ is essential for catalysis [10].

Due to its complexity, the F-ATP synthase assembly occurs in a modular fashion to prevent formation of intermediates that could depolarize the membrane or waste ATP [23], although the pathways are still debated [24]. The structural and functional coupling between F_O_ and F_1_ in the mature complex is mainly guaranteed by the OSCP or *δ* subunit in mitochondria and in bacteria, respectively. Through its contacts with both the *α*_3_*β*_3_ hexamer and the peripheral stalk, OSCP or *δ* prevents corotation of the *αβ* dimers with the *γ* subunit, thereby ensuring very high enzyme efficiency [25]. Another domain crucial for the functional coupling is located at the C-terminus of *β* subunit, which is in direct contact with *γ* subunit, termed the DELSEED loop, which is thought to transfer the torque to *γ* from the nucleotide binding domain [26]. Moreover, in mitochondria, the high catalytic efficiency of F-ATP synthase seems to be mediated also by formation of the V-shaped dimers [27, 28]. The electron cryomicroscopy maps of the mammalian [29] and yeast [18] enzymes revealed that subunits *e* and *g*, with the N-terminal part of subunit *b* and probably of subunit *k* in yeast, create in the F-ATP synthase monomers a subdomain that bends the IMM. These bends would drive self-assembly of the monomers into V-shaped dimers. Then, dimers self-assemble into long rows of oligomers localized at the cristae ridges [30] to maintain the typical IMM morphology [31]. The mitochondrial cristae would act as proton traps, favouring effective ATP synthesis by F-ATP synthase localized at the apex. Electron cryotomography showed that the dimers are organized in situ with the peripheral stalks turned away from one another [32] and are formed through the contribution of several F_O_ subunits (*a*, *b*, *e*, *f*, *g*, *i/j*, and *k*) [18].

## 3. Mitochondrial Uncoupling

**Figure 2:**
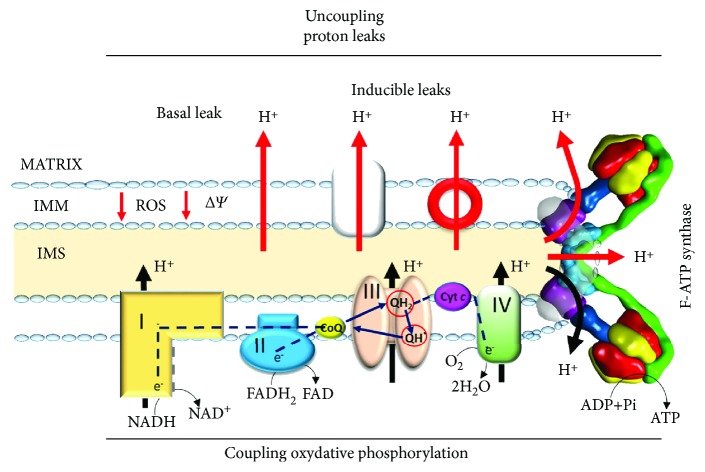
The proton circuit across the inner mitochondrial membrane. During the oxidative phosphorylation, the redox reactions of the four respiratory chain complexes are indirectly coupled to ATP synthesis by the F-ATP synthase dimers through the electrochemical proton gradient across the IMM. Return of protons into the matrix independent of ATP synthesis through the basal leak pathway, or through inducible leaks, decreases the electrochemical proton gradient and leads to mitochondrial uncoupling protecting mitochondria against ROS production. Proton back-leaks through the F_O_ sector of F-ATP synthase, independent of the synthesis of ATP, lead to dissipation of the proton gradient, thus transforming F-ATP synthase into an energy-dissipating structure. Red arrows correspond to proton leaks.

Mitochondrial uncoupling is quite a general term and refers to any pathway that enables proton reentry into the matrix independent of ATP production. Indeed, the oxidative phosphorylation involves the coupling of redox reactions of the respiratory chain to ATP synthesis by the F-ATP synthase through a proton cycle across the IMM. These reactions, however, are not fully coupled, since protons can return to the mitochondrial matrix independent of ATP synthesis by either unregulated endogenous pathways, termed basal proton leaks, which are indeed modifiable by drugs or by inducible leaks through protein complexes (Figure 2). This energy-dissipating cycling occurs in all eukaryotic cells and accounts for a varying proportion of cellular metabolism, depending on the tissue type [33].

Physiological uncoupling is typically mediated in mammals by the finely regulated uncoupling protein 1 (UCP1), an integral membrane protein of the brown adipose tissue (BAT) that mediates the leak of protons across the IMM, dissipating the proton gradient and inducing heat production [34]. Physiological uncoupling also enables fine-tuning of insulin secretion by uncoupling protein 2 (UCP2) in pancreatic *β* cells [35] and regulation of fatty acid metabolism by uncoupling protein 3 (UCP3), which is specific to skeletal muscle, BAT, and heart, although UCP2 and UCP3 functions are not clearly established [33]. Moreover, multiple ATP-dependent dissipative pathways exist in yeast mitochondria that include both selective [36, 37] and unselective channels [38]. In plants, besides UCPs [39], a further energy dissipation system has been described, called alternative oxidase (AOX), which couples ubiquinol oxidation with direct reduction of oxygen to water [40].

Mitochondrial uncoupling can also be induced by chemical uncouplers, i.e., small molecules that lessen the proton motive force across the IMM. These molecules belong to one of two general classes: protonophore uncouplers and nonprotonophores. Protonophore uncouplers, such as carbonyl cyanide p-trifluoromethoxyphenylhydrazone (FCCP), are lipophilic weak acids able to traverse the membrane as an uncharged form, inducing complete uncoupling at very low concentration, i.e., in the nM range, and causing sudden increases of the respiratory rates that are oligomycin-insensitive [41]. Nonprotonophores can instead activate latent proton leaks to variable degrees through specific protein complexes that lead to mitochondrial dysfunction. Mitochondrial uncoupling can be measured directly as a decrease in the electrochemical gradient or indirectly, as a decrease in the phosphorylation efficiency, i.e., ADP/O stoichiometry, or/and as a decrease of the respiratory control ratio (RCR), i.e., the ratio between oxygen consumption during (state 3) and after (state 4) ADP phosphorylation.

Mitochondrial uncoupling is not completely harmful. Indeed, there is a close inverse relationship between increasing the proton leak and ROS generation in isolated mitochondria [42]. It is long known that addition to isolated mitochondria of uncouplers, as well as ADP, increases the oxygen consumption and lowers the mitochondrial electrochemical gradient, decreasing ROS production [43]. Conversely, inhibition of the ATP synthase capacity leads to accumulation of the electrons in the upstream complexes of the respiratory chain, promoting oxidative stress and mitochondrial dysfunction [3], as recently observed in diabetic cardiomyopathy in mice [44]. These observations support a role for endogenous mitochondrial uncoupling in protection against ROS production [2, 33]. Consistently, therapeutic mitochondrial uncoupling is reported to be protective in a variety of disorders, including obesity [45, 46], diabetes [47, 48], ischemia/reperfusion injury [33, 49], Parkinson's disease [50], and aging [51], although the responsible factor(s) remain to be fully understood. Mild uncoupling can be obtained in different ways, i.e., by UCP activation, such as in obese diabetic mice, where UCP2 overexpression restored the impaired endothelium-dependent relaxation [48], or by the use of chemical uncouplers, such as niclosamide ethanolamine, which improved diabetic symptoms in mice [47], or the novel mitochondrial uncoupler BAM15, which has no off-target activity on other cellular membranes [52, 53]. Nevertheless, caution is always required when targeting mitochondrial uncoupling via lipophilic weak acids, even when they are selective, since, differently from the UCPs, their activity lacks autoregulation, i.e., is not desensitized by reduction of the membrane potential [7].

## 4. F-ATP Synthase Uncoupling

The term F-ATP synthase uncoupling refers to any condition that inhibits the coupling between the F_1_ catalytic activities and the proton translocation by F_O_. Conditions that activate the formation of a proton back-leak through F_O_ independently from the synthesis of ATP lead to dissipation of the proton gradient, thus transforming F-ATP synthase into an energy-dissipating enzyme. Indeed, the enzyme appears to have an intrinsic robustness, as recently demonstrated by the formation of stable incomplete subcomplexes after disruption of individual human genes for subunits in the F_O_ membrane portion [24]. However, the dissipative pathways and their modulation are still to be defined in the majority of cases. For example, mammalian mitochondrial oxidation of mono- and dithiols located in F_O_ induces a complete uncoupling of ATP synthase, which is not reverted by oligomycin [54]. The authors proposed that the lesion was on the cytosolic side of the oligomycin block point of the proton channel, a location that would suggest the involvement of the unique conserved cysteine residue of the subunit *c*. Moreover, the formation of a disulphide bridge between two vicinal subunits *b* of two adjacent F-ATP synthase monomers induces a severe oligomycin-insensitive uncoupling, of which the molecular mechanism has not been fully elucidated [55]. Another latent oligomycin-insensitive proton-translocating pathway in F_O_ would comprise the subunits *e*, *f*, *g*, and A6L, of which conductance was markedly increased upon displacement of the matrix protein factor B from F_O_. This effect is due to oxidation of vicinal thiols of factor B and is parallel to mitochondrial uncoupling [56, 57]. More recently, the matrix protein Bcl-XL has been identified in neurons as able to revert ATP synthase uncoupling by binding to the *β* subunit, although the underlying mechanism remains to be established [58, 59]. Interestingly, the leucine-rich pentatricopeptide repeat containing protein (Lrpprc), a key posttranscriptional regulator of mtDNA expression defective in the French Canadian type of Leigh syndrome, has also resulted to be crucial for F-ATP synthase coupling by modulating the proper assembly of the subunits OSCP and A6L. In the Lrpprc conditional knockout mouse heart, decrease in ATP production is due to the appearance of uncoupled subassembled F-ATP synthase complexes, causing hyperpolarization and increase of mitochondrial ROS production, in spite of an unaltered ADP/O ratio, thus showing the consequences of F-ATP synthase assembly defects on mitochondrial bioenergetics [60].

F-ATP synthase uncoupling is also stimulated by a number of cationic dyes, namely, coriphosphine, Nile blue, pyronin Y, and acridine orange, which increase both state 4 respiration and ATPase activity, but the binding sites remain to be established [61]. Another molecule able to stimulate a proton back-leak through the eukaryotic F_O_ is 17*β*-estradiol that, at micromolar concentrations, induces an “intrinsically slipping state” of F-ATP synthase, while the enzyme is actually catalysing ATP synthesis, therefore resulting in a depressed RCR and ADP/O ratio [62]. Such partially uncoupled state is promoted by ATP and reversed by oligomycin, but not by resveratrol, supporting the fact that the F_O_ moiety is a site of action of 17*β*-estradiol. Possibly, a conformational change is transmitted to F_O_ through the OSCP subunit, which contains a binding site for 17*β*-estradiol able to mediate the inhibition of ATPase activity both at nanomolar and micromolar concentrations [63, 64]. The ability of ATP to cause enzyme uncoupling seems conserved, since a transition from a tightly coupled to a loosely coupled state triggered by ATP binding is also described for bacterial ATP synthase, although the underlying mechanism remains to be elucidated [65, 66]. A complete uncoupling is instead induced by addition of Ca^2+^, which, differently from Mg^2+^, only sustains ATP hydrolysis by F_1_ that is not coupled to generation of a proton gradient in both prokaryotes [67] and eukaryotes [68–70], in spite of Ca^2+^ ability to sustain the F_1_ rotational catalysis [71]. These data strongly suggest that the catalytic site has a different conformation state when occupied by Ca^2+^ that, when compared with Mg^2+^, is unable to couple the chemical catalysis to the generation of a proton gradient.

Based on these observations, it has been suggested that Ca^2+^ binding, possibly by replacing Mg^2+^ at the catalytic site [72], together with ROS could cause a drastic conformational change to the ATP synthase dimer. This would give rise to the formation of a high conductance channel, named permeability transition pore (PTP), thus representing an extreme form of enzyme uncoupling. The PTP is a nonselective channel modulated by Ca^2+^ and ROS and is located in the IMM, and its opening implies the dissipation of the proton gradient with cessation of ATP synthesis and maximization of ATP hydrolysis. The PTP displays a range of conductance states, which have been originally characterized by electrophysiology in mammals [73]. Persistent PTP opening causes equilibration of low-molecular-weight (<1500 Da) molecules across the IMM, which disrupts any metabolic gradient, followed by an osmotic stress leading to matrix swelling and, eventually, to OMM rupture and release of proapoptotic factors like cytochrome *c*, endonuclease G, and AIF. Indeed, the PTP is causally involved in cell death in several diseases, and the most documented cases include heart ischemia, muscular dystrophies, and neurodegenerative diseases [74].

The involvement of F-ATP synthase in PTP formation has been supported by (i) genetic manipulation of selected enzyme subunits whose ablation affected the PTP function [75, 76]; (ii) electrophysiological measurements, which showed that the PTP generated by F-ATP synthase is characterized by a variety of conductances similar to those of the native pore [77, 78]; and (iii) mutagenesis of specific residues of F-ATP synthase within F_1_ [72] or F_O_ [79–81]. In particular, OSCP, located on top of F-ATP synthase, appears ideally suited to transmit Ca^2+^-dependent conformational changes from F_1_ to the rigid peripheral stalk causing pore formation within the F-ATP synthase membrane portion, probably at the interface between the two monomers forming an F-ATP synthase dimer. Indeed, this model is strongly supported by the observations that OSCP (i) is the binding site of cyclophilin (CyP) D, the best characterized PTP inducer, which sensitizes the PTP to the effects of matrix Ca^2+^ and is released by cyclosporin A resulting in PTP inhibition [77, 82]; (ii) is the binding site of the immunomodulatory drug benzodiazepine (Bz) 423 [83], which like CyPD acts as a PTP inducer [77]; (iii) contains a highly conserved histidyl residue (H112 in the human mature protein) responsible for the inhibitory effect of acidic matrix pH on the PTP [81]; (iv) undergoes, in contrast to major subunits of the ATP synthase complex, a selective decrease of its expression level, which is concomitant with CyPD upregulation and PTP activation in brain mitochondria from aging mice [84]; and (v) interacts with the *β* amyloid protein in cultured neurons, leading to PTP sensitization, and in the brain of individuals with Alzheimer's disease, potentially mediating mitochondrial impairment [85]. Nevertheless, the Ca^2+^- and ROS-dependent long-range conformational changes that could be responsible for the PTP formation in the F_O_ sector remain to be defined. Moreover, other models of PTP have been advanced that hypothesize the PTP resides in the *c*-ring of ATP synthase [76, 79] or, alternatively, in some other mitochondrial components not involving ATP synthase [86] so that its molecular nature is still a matter of debate [25].

## 5. F-ATP Synthase as a Target for Drug Development

Until now, malfunction of F-ATP synthase has been associated with a variety of pathological conditions, such as cardiovascular [87] and neurodegenerative diseases [88], obesity and type 2 diabetes [89, 90], and cancer [91]. Despite such important evidence, F-ATP synthase has only recently been used as an effective drug target for disease conditions and for the regulation of energy metabolism [92], despite the fact that more than 300 natural and synthetic molecules are known to bind and inhibit this complex [93]. Moreover, the recent finding that F-ATP synthase is involved in PTP formation may make this complex a viable target for future therapy in a variety of diseases [25]. Selective interaction of F-ATP synthase with some drugs has been identified. For example, the enzyme is recognized as a molecular target of the cytotoxic agent apoptolidin, which induces apoptosis in human cancer cell lines by binding to the F_O_ subunit *a* and inhibiting the enzyme activity [94]. Another example is provided by the immunomodulatory drug Bz 423, which activates the mitochondrial pathway of apoptosis selectively in pathogenic lymphocytes [83]. More recently, the *α* subunit of the F_1_ sector has been recognized as a target for the drug candidate J147, which shows therapeutic efficacy in several mouse models of Alzheimer's disease. By targeting ATP synthase, J147 causes an increase in intracellular Ca^2+^ leading to activation of the AMPK/mTOR pathway, a canonical longevity mechanism [95]. Moreover, the enzyme is a very promising molecular target for the development of new antimicrobial agents that selectively inhibit the bacterial F-ATP synthases, such as bedaquiline that represents the first compound of a new class of potent antituberculosis drugs [96]. Attention has recently focused on dietary phytochemicals with antimicrobial properties, a variety of which inhibits the bacterial F-ATP synthase to a variable degree depending on the type and positioning of the functional groups. Both F_1_ and F_O_ subunits have been identified as contributing to the binding sites for such inhibitors [92]. Because dietary phytochemicals also inhibit the mitochondrial F-ATP synthase [97], attempts are being made to modify the functional groups of these compounds, making them more potent and selective inhibitors of the bacterial ATP synthases [92]. On the other hand, many of these phytochemicals exhibit diverse activities, such as antioxidant, anticancerogenic, and antiobesity actions [97]. An example is resveratrol, a well-characterized inhibitor of the mitochondrial F-ATP synthase whose binding site is located between the C-terminal part of the *γ* subunit and the *β*_TP_ subunit [98]. Resveratrol also increases the basal energy expenditure and thermogenesis, along with alteration of numerous signalling pathways that converge on the mitochondria [99].

Recently, there has been an increased interest in the use of essential oils (EOs) as preventive and therapeutic agents for treatment of various diseases, including obesity [100]. EOs are secondary metabolites of plants representing a mixture of a variety of volatile molecules such as terpenoids and phenol-derived aromatic and aliphatic components. EOs also possess antimicrobial, anti-inflammatory, anticancer, and antioxidant properties, but the molecular targets are still to be defined in the majority of cases [101]. The EO component D-limonene, which is recognized as a potential chemotherapeutic agent [102], induces apoptosis via the mitochondrial pathway in several human cell lines [103] and has also antiobesity activity, mainly by induction of the brown fat-like phenotype in white adipocytes [104]. Interestingly, the EO component *p*-cymene directly induces a mild uncoupling of F-ATP synthase, leading to an ATP-stimulated, oligomycin-sensitive proton leak through the F_O_ moiety that decreases the electrochemical gradient and the respiratory control ratio but not the ADP/O ratio [105]. Such properties might suggest its use as a pharmacological agent to decrease the metabolic efficiency. However, caution is required, because affecting all mitochondria throughout the body may be a high-risk treatment, as energy homeostasis may be compromised in tissues such as the heart and brain.

## 6. Conclusions

The existence of several conditions that inhibit or abolish the coupling between F_1_ and F_O_ clearly indicates that the energy-conserving enzyme can transform into an energy-dissipating structure within the IMM. Available structures of the fully coupled F-ATP synthase do not display obvious features that can accommodate these dissipative pathways. Indeed, the molecular definition of the pathways responsible for F-ATP synthase uncoupling and of its potential regulators still represents a research challenge in bioenergetics. Due to the central role of F-ATP synthase in cellular metabolism, this definition is crucial and potentially useful for therapy in a variety of diseases.
